# Rheological and Aging Properties of Composite Modified Bitumen by Styrene–Butadiene–Styrene and Desulfurized Crumb Rubber

**DOI:** 10.3390/polym13183037

**Published:** 2021-09-08

**Authors:** Gang Xu, Yunhong Yu, Jingyao Yang, Tianling Wang, Peipei Kong, Xianhua Chen

**Affiliations:** National Demonstration Center for Experimental Road and Traffic Engineering Education, School of Transportation, Southeast University, Southeast University Road, Nanjing 210096, China; xugang619@hotmail.com (G.X.); iiyyhong@163.com (Y.Y.); yangjingyao19@foxmail.com (J.Y.); 220183061@seu.edu.cn (T.W.); xc_kong@seu.edu.cn (P.K.)

**Keywords:** desulfurized crumb rubber, styrene–butadiene–styrene, composite modified bitumen, rheological properties, aging process

## Abstract

Taking advantage of crumb rubber from waste tires to modify bitumen is widely for the environmentally friendly and sustainable development of pavement. This study investigated the modification mechanism, rheological, and aging properties of styrene–butadiene–styrene (SBS)/desulfurized crumb rubber (DCR) composite modified bitumen (SBS/DCRMB). Morphological features and chemical characteristics were assessed by fluorescence intensity measurement and gel permeation chromatography (GPC), respectively, and results demonstrated that the DCR and SBS modifier in SBS/DCRMB had been vulcanized and formed a three-dimensional network structure. Moreover, a comparison of the GPC elution curve showed the residual bitumen hardly changed due to carbon black released from DCR of SBS/DCRMB during the aging process of SBS/DCRMB, and the polymer molecules condensed to larger units. However, the remaining bitumen in SBSMB had changed evidently and the polymer degraded to smaller molecules. Meanwhile the rheological testing results, including multiple stress creep recovery, linear amplitude sweep and bending beam rheometer, declared that the SBS/DCRMB is superior to SBSMB before and after aging.

## 1. Introduction

Nowadays, the noticeable increase in traffic volume and the dramatic increase in heavy vehicle axle load has led to the necessity to attention focus on pavement performance. At the same time, base bitumen cannot meet the requirements of the structure and function of bitumen concrete pavement [1,2]. To address this issue, more and more modified bitumens, such as styrene–butadiene–styrene (SBS) modified bitumen (SBSMB), have been applied to high-grade pavement, which significantly enhances the performance of bitumen pavement [3,4]. However, the high cost of SBSMB indicates its incapability to achieve resource conservation and environmental friendliness. Therefore, bitumen modified with crumb rubber (CR) prepared from waste tires has been considered by many researchers due to its advantages of energy thriftiness, environmental protection, and economic significance [5,6]. Currently, CR modified bitumen (CRMB) has been utilized as a binder of asphalt mixtures on many roads, both in China and internationally, which achieves promising performance and effectively reduced pollution of the tire. However, due to the three-dimensional structure of rubber molecules and vulcanization, it is difficult to swell during processing. Furthermore, crumb rubber rarely reacts with bitumen, resulting in rubber and bitumen prone to natural segregation, poor storage stability under high temperature, and other defects [7,8,9]. These issues restrict the further development of CRMB in road-building technology. Hence, researchers have been pre-treating the crumb rubber to improve the performance of CRMB.

It has been investigated that the desulfurization of crumb rubber could significantly improve the performance of CRMB. Desulphurization promotes the activity of crumb rubber, which can substantially improve the performance of rubber bitumen and its mixture [10,11,12]. As indicated by the literature, desulfurized rubber particles have a low crosslinking degree and more active chemical bonds, enabling more apparent chemical effects between the rubber particles and bitumen. Furthermore, the high-temperature viscosity is noticeably decreased, and the preparation temperature also decreased slightly. In addition, it is more environmentally friendly, as less odor exhausts during the preparation process [13]. Moreover, the solvents and other reactive agents are added in the preparation process of CRMB, which can ameliorate the phase dissolution rate of rubber and bitumen, as well as improve the stability of CRMB. Desulfurized crumb rubber modified bitumen (DCRMB) and reactive rubber modified bitumen exhibits high elastic response, which can reduce non-recoverable deformation, thus yielding a better low-temperature crack resistance and fatigue resistance [14,15,16,17].

Additionally, CRMB has increased flexibility and better resistance to heat and oxygen aging, and features many other beneficial properties compared with base bitumen. However, it has been proven that the rutting resistance and storage stability of bitumen modified by desulfurized rubber still remain poor. In this regard, it can be concluded that bitumen single modifier is hard to satisfy the multiple performance requirements of bitumen pavement [12,18,19,20]. Consequently, researchers began to combine two or more kinds of modifiers and methods for modifying bitumen, to combine the advantages different modifiers offer. For example, tire crumb rubber combined with recycled polyethylene extruded melt blend as a composite additive was conducted to enhance the performance of binder and results found recycled polyethylene played a key role to improving the high-temperature performance of composite modified bitumen [21].

SBS copolymers are widely used for bitumen modification. However, the main drawbacks of SBSMB are high cost and poor aging resistance. Hence, researchers began to examine the application of SBS/DCRMB, as not only can it unite the performance advantages of DCR and SBS, but it can also reduce the SBS modifier dosage to reduce construction costs as well as the environmental pollution caused by the utilization of crumb tires in bitumen [22,23,24]. According to the results of morphology observation, the addition of SBS to CRMB leads to the formation of a staggered polymer network. At the same time, vulcanization or aging would improve the compatibility between polymer and bitumen, enhances their interaction and increases the storage stability of SBS/DCRMB [25,26]. The results also indicate that carbon black will be released during the process, which can improve the flexibility of network structures and boosts their anti-aging property. Furthermore, black carbon increases the adhesion of bitumen, so as to improve the general performance of bitumen mixture [27,28,29]. In summary, the performance of the SBS/DCRMB has been improved over a wide temperature range, as the SBS in the bitumen enhances the high-temperature properties, DCR improves the fatigue and low-temperature cracking resistance due to its excellent elasticity. However, current studies on SBS/DCRMB were merely the simple addition of two single modifiers by different orders, where there is no further pretreatment of the two types of modifiers. In addition, detailed descriptions about the morphology and the systematic discussion of the combined modification mechanism of the two modifiers are still unclear.

In this sense, this research proposes a type of SBS/DCRMB by SBS/DCR composite additive, which was prepared by force-chemical reactor. The modification mechanism of SBS/DCRMB was characterized by fluorescence intensity measurement and gel permeation chromatography (GPC). The rheological properties, including rutting, fatigue, and low-temperature performance of SBS/DCR and SBS modified bitumen were analyzed and compared before and after thin film oven test (TFOT) and pressurized aging vessel (PAV) aging. Based on the results of GPC, the relationship between the molecular distribution and macroscopic properties was explored. Then, their ability to resist aging was compared between SBS/DCRMB and SBSMB.

## 2. Methods

### 2.1. Materials and Preparation

**Figure 1:**
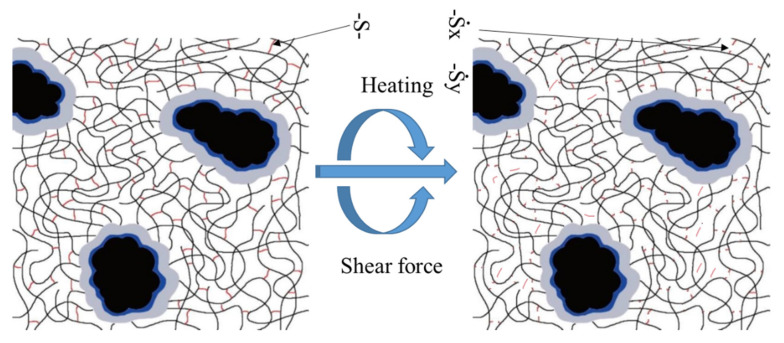
The principle of desulphurization by twin-strew extruder.

Pen70 was selected as the neat binder for modified bitumen, and the physical properties are shown in Table 1. Two conventional polymers were selected, including SBS and desulfurized crumb rubber (DCR). The average molecule weight of linear SBS was 80,000 g/mol, and the weight percentage of styrene was 30%. The DCR modifier was devulcanized by twin-strew extruder and the principle of desulphurization is shown in Figure 1. Meanwhile, rubber processing oil, which is rich in aromatics and saturates, was utilized to achieve sufficient DCR and SBS swelling in bitumen. The content of rubber processing oil was 4% by mass of base bitumen. Sulfur powder acted as crosslinking agent, and an amount of 5% of the modifiers weight was applied.

**Figure 2:**
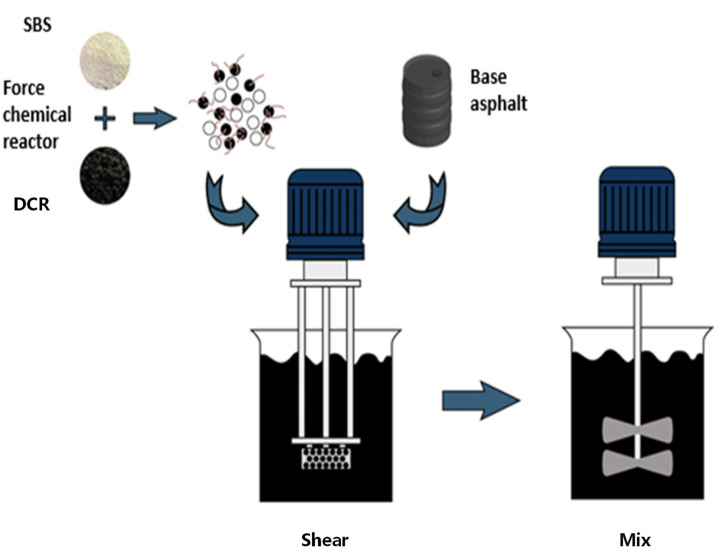
Flowchart of material preparation.

DCR/SBSMB was prepared in a laboratory at 180 °C. Firstly, the DCR/SBS composite modifier was added to the base asphalt and the blend was sheared at a speed of 6000 r/min for 1.5 h. The speed was then reduced to 1500 r/min for 1h until fully swelled. The preparation process of the DCR/SBSMB is illustrated in Figure 2. Conventional CR/SBS composite modified asphalt with 20% CR and 4% SBS was prepared using the same method.

As mentioned above, there was a type of bitumen considered as references for the comparison of SBS/DCRMB, namely SBSMB with 4% SBS (of the weight of base bitumen). It should be noted that the afore-mentioned preparation conditions were also used for preparation of SBSMB.

### 2.2. Aging Method

The bitumen samples were assessed using TFOT aging at 163 °C for 5 h according to ASTM D1754 to simulate the short-term aging of bitumen during mixing, transportation, and paving [35]. Furthermore, long-term aged bitumen was obtained by conducting TFOT (for 5 h at 163 °C), followed by a PAV test. ASTM D6531 recommends imitating the age of the road’s service life [36]. The aging temperature in the PAV test was 100 °C. The pressure was set to 2.1 MPa and the aging time was 20 h.

### 2.3. Fluorescence Intensity Measurement

Fluorescence microscopy is a standard method to observe the state of asphalt binders during their modification with polymers [37,38]. The polymer-rich phase that is swelled by the oily aromatic part of the asphalt appears to be yellow-colored, while the asphaltene phase is dark [39,40]. A Nikon Y-IDP Japan fluorescence microscope (Nikon, Tokyo, Japan) was employed to measure the fluorescence intensity of the modified bitumen, which supports up to 400× *g*. Under fluorescence, when excited by short-wave light, the polymer phase, formed by the swelling of the polymer, will emit a longer wavelength of light, while the asphalt phase emits no light. Therefore, it is easy to distinguish the yellow polymer phase from the black asphalt phase under a fluorescence microscope, and the distribution of modifiers in the asphalt can be clearly seen.

### 2.4. Molecular Distribution Analysis Using Gel Permeation Chromatography

GPC (HLC-8320, Tosoh, Tokyo, Japan) was employed to characterize changes of the molecular dimension. A total of five columns (TSK gel Super HM-M W0054, TSK gel Super HM-M W0055, TSK gel Super HM-M W0056 TSK gel Super H-RC W0109, and TSK Guard Column Super HH W0114, Tosoh, Tokyo, Japan) are required for GPC to separate asphalt binders based on their molecular size. Before the test, tetrahydrofuran was used to dissolve a small amount of asphalt to prepare a 0.5–5 mg/mL solution. The THF flow rate was controlled at 0.2 mL/min. After the test, software configured by a GPC device was used to calculate the number average molecular weight (Mn) and weight average molecular weight (Mw). Then, their ratio was used to represent the molecular distribution length and investigate the molecular distribution of modified asphalt before and after aging [29,41,42].

### 2.5. Multiple Stress Creep Recovery Test

The multiple stress creep recovery (MSCR) test was conducted by a dynamic shear rheometer (DSR) device ( Anton-paar, Graz, Austria) based on AASHTO T350 [43]. The diameter of samples used for testing was 25 mm, the thickness was 1 mm and the testing temperature was 64 °C, which can simulate the anti-rutting performance. The results of the MSCR test were obtained based on two replicates. The MSCR test consisted of 20 cycles, a 1 s creep period, and a 9 s recovery period at a stress level of 0.1 kPa. This was followed by another 10 cycles of creep and recovery at 3.2 kPa according to AASHTO T350. The three main parameters obtained in the experiment mainly used the following Equations (1)–(3).

(1) $$\mathrm{Recovery}\%\;\mathrm{at}\;100Pa\;\mathrm{or}\;3200Pa=\frac{1}{10}\left[\overset{10}{\underset{i=1}{\sum }}\frac{\gamma_{\left(r\right)i}}{\gamma_{\left(t\right)i}}\right]\times 100$$

(2) $$J_{nr\;at\;100Pa}\left(1/KPa\right)=\frac{1}{10}\left[\overset{10}{\underset{i=1}{\sum }}\frac{\gamma_{\left(nr\right)i}}{0.1}\right]$$

(3) $$J_{nr\;at\;3200Pa}\left(1/KPa\right)=\frac{1}{10}\left[\overset{10}{\underset{i=1}{\sum }}\frac{\gamma_{\left(nr\right)i}}{3.2}\right]\;$$

where $\gamma_{\left(r\right)i}$ is recoverable strain, $\gamma_{\left(nr\right)i}$ is non-recoverable strain at the end of 9 s rest, and $\gamma_{\left(t\right)i}$ is total creep strain at the end of the loading time of 1 s in each cycle.

### 2.6. Linear Amplitude Scanning Test

Current asphalt binder specifications lack the ability to characterize the damage resistance of asphalt binder to fatigue loading. Multiple accelerated testing procedures are currently being investigated in an attempt to describe the contribution of asphalt binders to mixture fatigue efficiently and accurately [17,40]. One of these tests, which has received acceptance by experts and has been submitted as a draft AASHTO standard, is the linear amplitude sweep (LAS) test. The LAS test was conducted at 25 °C. According to AASHTO TP 101-14, the asphalt samples (8 mm in diameter and with a 2-mm thickness) were tested in two stages [44]. In the first stage, a frequency sweep test with a strain level of 0.1% was performed at different frequencies (0.2 to 30 Hz). This test was used to obtain the undamaged material parameter (α). At the second stage, the oscillating shear was used at a frequency of 10 Hz in strain control mode at the selected temperature. The scanning time was 300 s, and the loading amplitude increased linearly from 0.1% to 30%.

### 2.7. Bending Beam Rheometer Test

The bending beam rheometer (BBR) test was employed to characterize the low-temperature performance of SBSMB and SBS/DCRMB before and after aging. The test temperatures were −12, −18, and −24 °C, and the average results of three replicates were used as the testing results. The two key parameters of bitumen at low temperature, as obtained by Equations (4) and (5), respectively, are creep stiffness modulus (S) and creep rate (m).

(4) $$S\left(t\right)=\frac{PL^{3}}{4bh^{3}\delta \left(t\right)}\;$$

(5) m=a+2blg(t) 

where S(t)(MPa) is flexural creep stiffness and P(N) is constant load. *h, L*, and *b* (mm) are thickness, length, and width of a thin beam sample, respectively. δ(t)(mm) is deformation at the mid-span of a thin beam sample.

Using the interpolation method according to ASTM D7643-16 can determine the low service temperature (*T_L_*) of asphalt binders from BBR test in multiple testing temperatures [45]. The critical temperature *T_L_,s* and *T_L_,m* corresponding to stiffness = 300 MPa and m value = 0.3 were obtained by the regression Equations (6) and (7), respectively. The low service temperature (*T_L_*) was defined as Equation (8).

(6) $$\log_{10}\left(s\right)=a_{1}+b_{1}T$$

(7) $$m=a_{2}+b_{2}T$$

(8) $$T_{L}=\max\left(T_{L,s},T_{L,m}\right)-10$$

where $a_{1}$, $a_{2}$, $b_{1}$, and $b_{2}$ are the regression coefficients; T is the test temperature (°C); $T_{L,s}$ is the critical temperature when S = 300 MPa (°C); $T_{L,m}$ is the critical temperature when m = 0.3 (°C); and $T_{L}$ is the low service temperature (°C).

## 3. Results and Discussion

### 3.1. Fluorescence Microscopy (FM) Analysis of SBS and DCR in Bitumen

**Figure 3:**
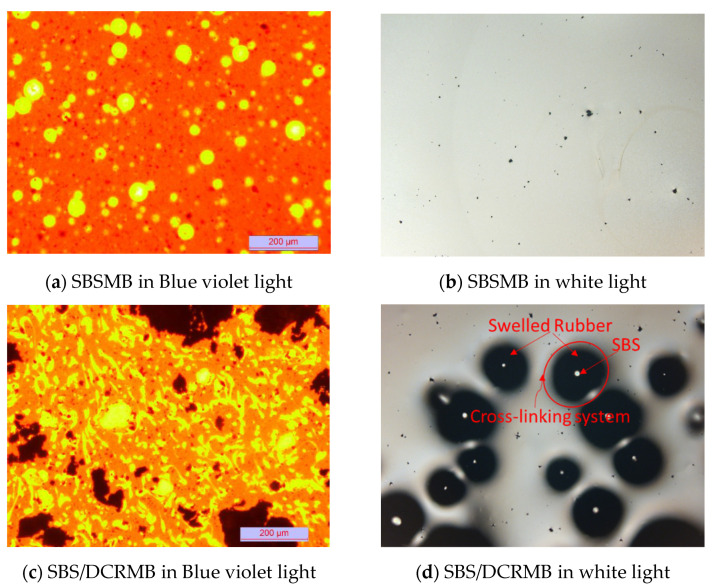
Micrography of SBSMB (**a**,**c**)and SBS/DCRMB (**b**,**d**) with different magnifications and different light sources in Fluorescence.

The fluorescence micrographs of SBSMB and SBS/DCRMB are shown in Figure 3. Figure 3a shows that under the excitation of violet light, the yellow spots are SBS components, and the orange parts are asphaltenes. The dot interface of SBS is clear and has a poor affinity with asphalt, resulting in poor compatibility. However, Figure 3c presents more black carbon components, and an irregular distribution shape of SBS. The interface is fuzzy and the interaction with asphalt is enhanced, thereby increasing the stability of the interface.

The fluorescence microscopic images of SBSMB and SBS/DCRMB are different under white light. As illustrated in Figure 3b, a few black spots (which might be impurities in the bitumen) were scattered in the white setting (base bitumen), while in Figure 3d, clear white dots are either surrounded by or connected to black patches. Comparing Figure 3b,d indicates that the aromatic component of bitumen or rubber processing oil is absorbed by DCR and the border of black patches are blurred and thick. Due to the addition of crosslinking agents during the preparation, SBS and DCR are crosslinked by non-directional vulcanization. In the process of DCR swelling, SBS is wrapped up between the DCR, and the polymer network structure of SBS/DCR forms [46].

**Figure 4:**
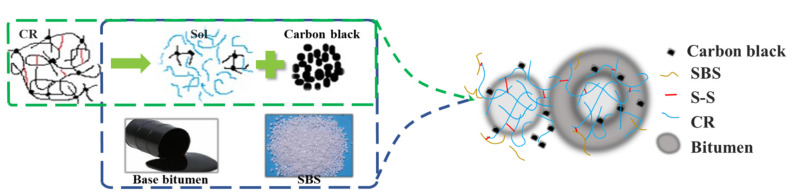
Modification mechanism of SBS/DCRMB.

These results indicate that during the preparation of SBS/DCRMB, aromatic components and rubber processing oil are absorbed during swelling, depolymerization, and dispersion. Under the interaction with sulfur, SBS, DCR, and the SBS–DCR polymer network, i.e., the cohesive forces between polymer, as shown in Figure 4, which briefly present the mechanism of composite modified bitumen.

### 3.2. Molecular Distribution Analysis with GPC

GPC analysis can effectively characterize the molecular structure and the decomposition of polymers during the aging process [42]. According to Figure 4, two regions can be observed from the GPC chromatogram of SBS/DCRMB and SBSMB. These are the residual matrix bitumen region on the left (with smaller molecular weight) and the polymer region on the right (with larger molecular weight). The appearance of the two molecular weight regions in SBS/DCRMB indicates that a mixed crosslinking system formed between SBS and DCR. The existence of a crosslinking system is verified via fluorescence microscopic images under white light.

**Figure 5:**
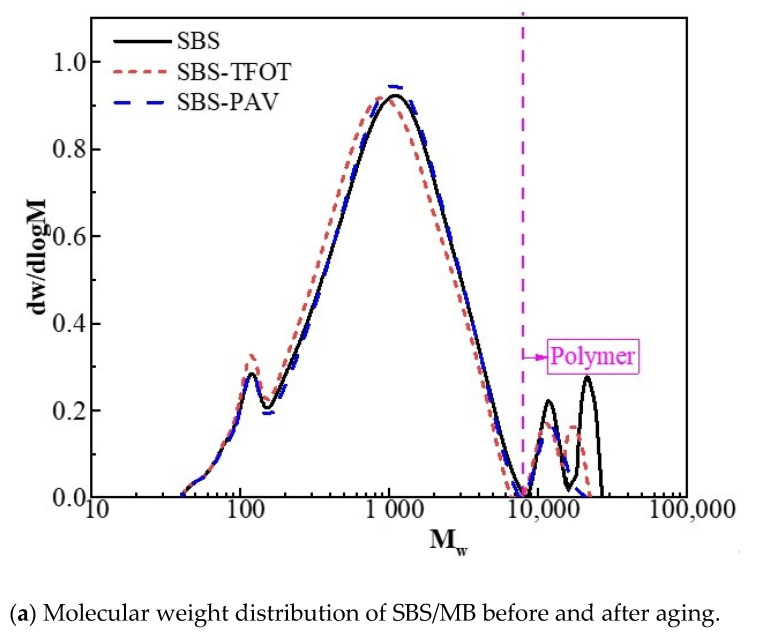

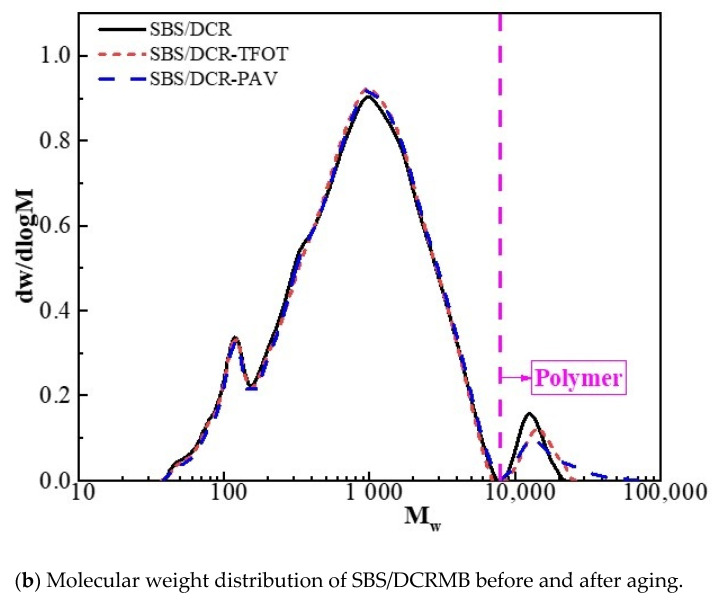
Molecular distribution of SBSMB and SBS/DCRMB in different aging conditions.

Focusing on the left region, Figure 5a shows that the aging effect leads to significant differences among the three peaks of the molecular weight distribution of residual asphalt. In contrast, the SBS/DCRMB residual bitumen molecular weight distribution curve shown in Figure 5b approximates an overlap and with good consistency. This may be ascribed to the following two reasons: On the one hand, the aromatics component is absorbed by the swelling of polymer components during the preparation of SBS/DCRMB. On the other hand, the residual bitumen is protected by the black carbon released during the preparation of DCR from thermo-oxidative aging.

Furthermore, focusing on the right region, the results of GPC also show the significant variation trend of the polymer region during the aging process. In SBSMB, the polybutadiene chains in SBS were destroyed, and the polymer molecular weight decreased, which leads to a left shift of the molecular weight distribution curve and damages the crosslinking structure. Dissimilarly in the polymer area of SBS/DCRMB, in response to aging, causes the curve to move to the right, indicating that some chemical reactions (such as condensation) are caused between the two polymers (SBS and DCR). Condensation is another variation of the reaction of bitumen aging that will generate larger molecules.

Ultimately, Mn can be used to characterize the variation of small molecules, while Mw reflects the macromolecule. Table 2 presents the detailed values of Mn, Mw, and Mn/Mw obtained by GPC test. Given the amount of macromolecular component, it is conflicting that the value of Mw would decrease in response to the dissociation of polymer modifier. The value of Mw would increase by the condensation of polymer and the conversion of aromatic components. As the indicator of Mn is free from such conflict, Mn can adequately reflect the aging resistance of modified bitumen. According to the variation of Mn, the Mn value of SBS/DCRMB is higher than that of SBSMB before aging, while SBSMB is higher than SBS/DCRMB after PAV aging. This implies that SBS/DCRMB has a better anti-aging ability.

In addition, the ratio of Mn to Mw in Table 2 shows that the ratio of SBS becomes smaller. This corresponds to the polymer distribution in Figure 5a, where the dispersion of the molecular distribution becomes smaller due to degradation. However, the ratio of SBS/DCRMB follows an increasing trend, and there is a significant increase between short-term aging and PAV. This corresponds to the molecular weight distribution of the polymer part, which increases due to shown polycondensation in Figure 5b.

### 3.3. Multiple Stress Creep Recovery Results

**Figure 6:**
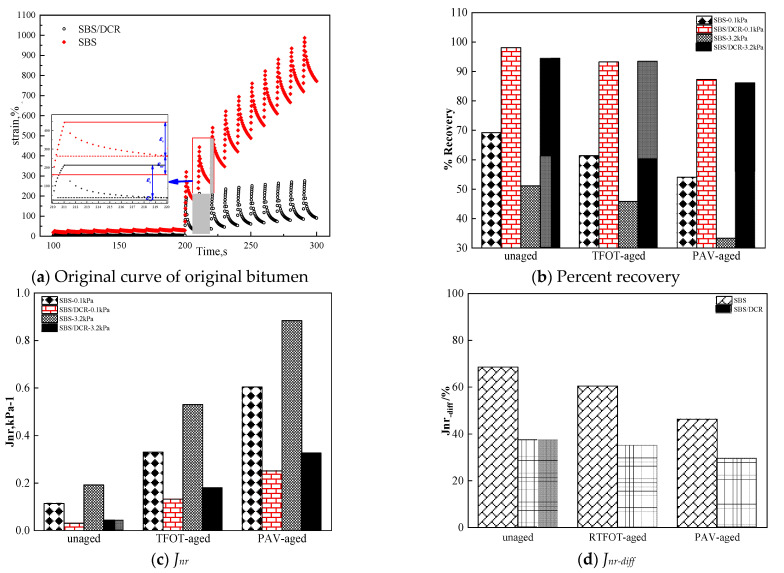
MSCR test result in different aging conditions of two modified bitumen.

Figure 6a shows the time–strain curve of un-aged SBSMB and SBS/DCRMB in a MSCR test. With increasing test time, the original bitumen strain also increased, and the strain of SBSMB became significantly larger than that of SBS/DCRMB. Additionally, the unrecoverable creep compliance ($J_{\mathrm{nr}}$) of SBS/DCRMB is much smaller than that of SBSMB. This illustrates that SBS/DCRMB possesses much higher resilience and resistance to the high-temperature deformation than SBSMB under the same loading conditions. The remarkable anti-rutting performance of SBS/DCRMB benefits from the high elastic property of DCR and the strong three-dimensional network structure developed by DCR and SBS.

Non-recoverable compliance ($J_{\mathrm{nr}}$), stress sensitivity ($J_{\mathrm{nr}-diff}$), and the creep recovery rate are the main parameters for evaluating the high-temperature properties of bitumen binders in MSCR testing [19,47]. Figure 6b–d present the results of the MSCR test for different aging conditions. All obtained results show differences between the two modified types of bitumen. These results show that the rate of creep recovery of SBSMB decreased significantly with increasing loading stress and aging, but the rate of creep recovery of SBS/DCRMB varies slightly. This might be related to the three-dimensional network structure formed by SBS and DCR during the modification process (as shown in the fluorescence micrographs). The complete three-dimensional network structure can be gradually recovered with the superior elastic properties provided from DCR when the stress diminishes. In contrast, the polymer network structure in SBSMB is difficult to rebuild due to a lack of elastic recovery and sensitivity to stress (as shown in Figure 3a).

However, the $J_{nr}$ of the two modified bitumens contrasts with the rate of creep recovery before and after aging. As shown in Figure 6c, $J_{nr}$ increases with increasing unrecoverable deformation at a higher stress level and with the degree of aging. This contrasts with the conclusion by Zhou et al., who claimed that the stiffness of base bitumen is enhanced after adding RAP, which resulted in a reduction in *J_nr_* [48]. After short-term and long-term aging, the modifiers decomposed as indicated by the GPC test. The decomposition of modifiers leads to the weakening of the polymer network structure and the interaction between the modifiers, thus reducing the resistance to deformation. Figure 6d shows the stress sensitivity of two types of bitumen under different aging conditions through *J_nr-diff_*. To a certain extent, the stress sensitivity of the modified bitumen is reduced by aging and all values were less than 75%. The GPC testing results of SBSMB indicate that in the process of thermo-oxidative aging, the aromatic component of SBSMB is reduced, and the stiffness is improved, thus, bitumen became insensitive to stress.

Based on the non-recoverable compliance, stress sensitivity, and the rate of creep recovery obtained by the MSCR test, the elastic properties of bitumen improved effectively by adding DCR. Additionally, as the polymer network formation by the DCR and SBS and the black carbon release by the DCR during the preparation process, the high-temperature performance of the bitumen was significantly improved, resulting in excellent road performance of the SBSMB.

### 3.4. Linear Amplitude Scanning Test Results

**Figure 7:**
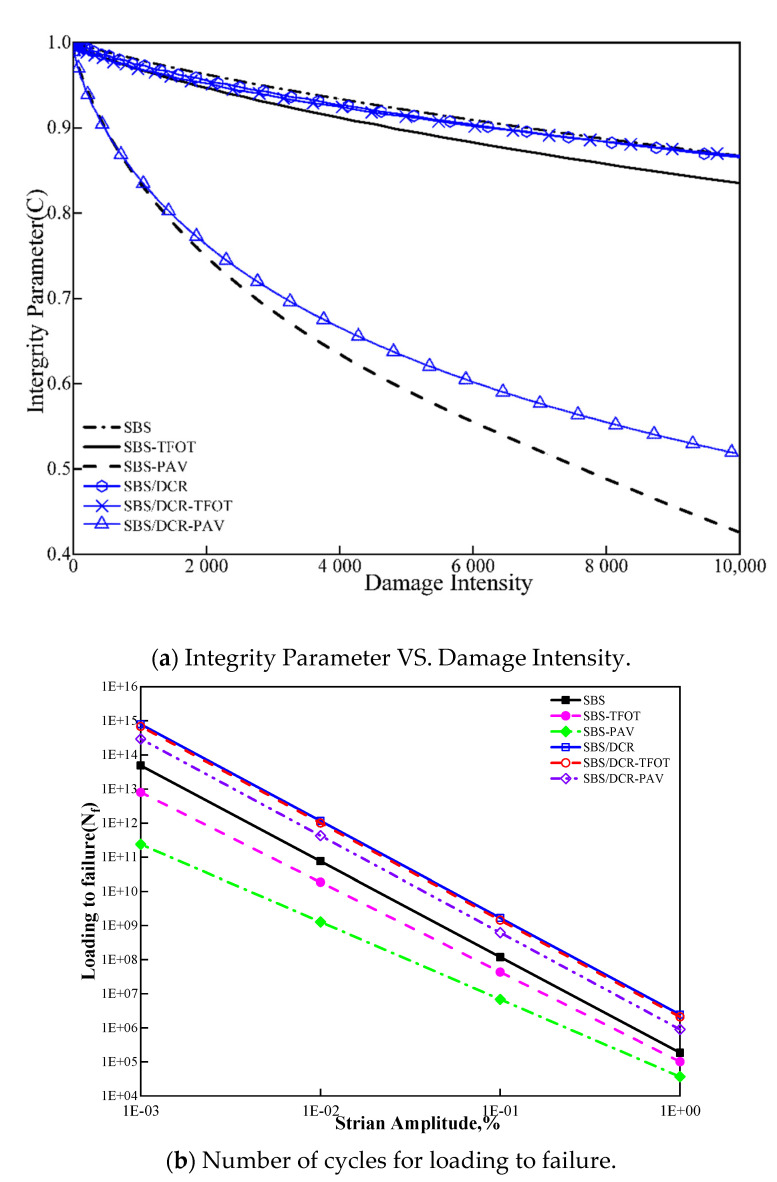
Results Linear Amplitude Scanning Test in Different aging conditions. (**a**): Integrity Parameter VS. Damage Intensity; (**b**): Number of cycles for loading to failure.

Table 3 displays the fatigue damage values obtained by LAS test based on the VECD model. Figure 7a,b present the rapid fatigue damage curve and the relationship between damage intensity (D) and integrity parameters (C), respectively. Table 3 shows that the C_1_ and C_2_ of SBSMB bitumen increase with aging, while the values of SBS/DCRMB show a completely different variation. Here, C_1_ decreases while C_2_ increases. The lower C1 and C2 values, the better the fatigue performance of bitumen will become [47]. During the aging process, the magnitude of the bitumen modulus, the increase in the polymer content, and the strength of the network will increase C_1_ and decrease C_2_. Combined with the results of GPC testing, the decomposition of the polymer and network structure during aging increases C_2_. For C_1_ parameters, in SBSMB, the positive effect from the increasing modulus is not counteracted by the weak or negative impact caused by the decomposition of the polymer. However, with the addition of DCR, the polymer proportion in SBS/DCRBM increases and the condition is improved. The impact of the damage intensity and the integrity parameter combined with C_1_ and C_2_ should be comprehensively considered.

As shown in Figure 7a, during the aging process, SBSMB is more sensitive to aging compared with SBS/DCRMB. During aging, the D–C curves of SBSMB indicated a large difference between before and after the same short-term aging, which is similar to SBS/DCRMB. Additionally, the loss rate of the integrity parameter increased sharply after PAV aging. The reason for this change may also be consistent with the result of the GPC test. In the aging process of SBSMB, a number of aromatic compounds are converted into asphaltenes, which play a dominant role in fatigue performance. Moreover, the degradation of modifier and the damage of the polymer network also accelerated the loss of fatigue performance. Therefore, aging severely impacts the fatigue performance of asphalt. SBS/DCRMB offers an advantage in resisting short-term aging. After PAV aging, the fatigue performance of asphalt cementation material decreases sharply. Therefore, the fatigue performance of asphalt is severely affected by aging and SBS/DCRMB offers an advantage in resisting short-term aging.

Figure 7b demonstrates that the loss rate of asphalt sensitivity decreases gradually with increase in strain level and aging degree. Comparing the loss of the fatigue life before and after aging shows that the fatigue life of SBS/DCR modified bitumen is longer than that of SBSMB bitumen regardless of the underlying aging conditions. This is caused by the addition of DCR, which can improve the fatigue resistance. It can also be found that aging conditions affect the bitumen fatigue life, and its effect is the same than that of the loss rate of the integrity parameter.

In conclusion, the effect of aging on the fatigue life of SBSMB is mainly the result of the hardening of bitumen. In contrast, the fatigue performance loss of SBS/DCRMB is primarily caused by polymer condensation and the disintegration of the structure of the polymer network.

### 3.5. Low-Temperature Performance

**Figure 8:**
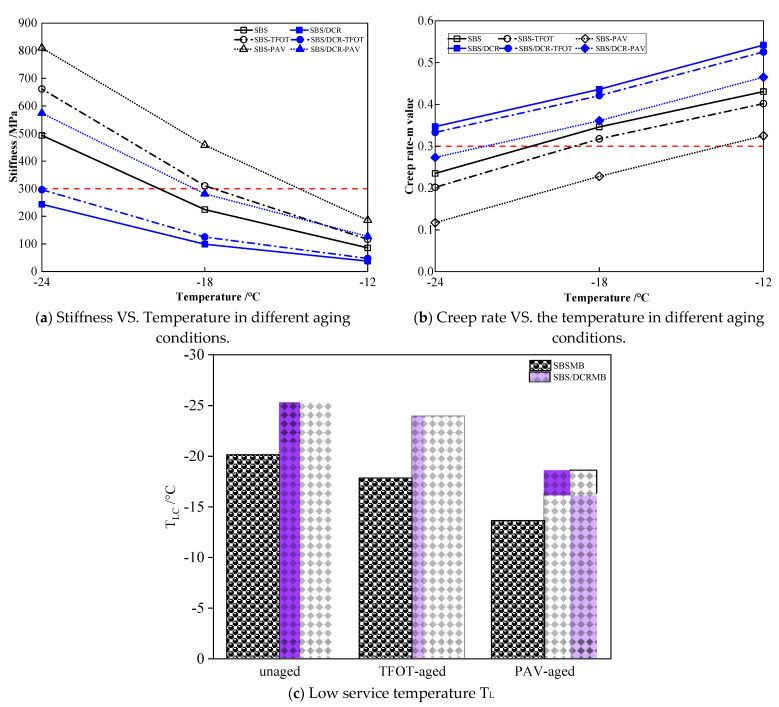
Low-temperature performance in bending beam Rheometer.

The change in stiffness modulus and the creep rate of SBSMB and SBS/DCRMB are presented in Figure 8a,b and the low service temperature (T_L_) of SBSMB, and SBS/DCRMB were compared in Figure 8c. The depicted results indicate that the stiffness modulus increased, and the creep rate decreased, after long-term aging at low temperature. Existing research indicates that low stiffness modulus and high creep rate are conducive to crack resistance, implying that the binder has better low-temperature performance. A comparison of the stiffness modulus and creep rate with temperature changes for different aging conditions shows that the degree of aging will increase the sensitivity of stiffness modulus and creep rate. Furthermore, regardless of the aging state, the S value of SBS/DCRMB is smaller than that of SBSMB at the same temperature, while the m value is larger. According to the AASHTO T313 standard, the minimum S value and the maximum m-value for PAV-aged asphalt binders should be 300 MPa and 0.3, respectively. From Figure 6, the stiffness values of SBSMB-PAV at 18 °C is larger than 300 MPa and m-value is smaller than 0.3, but SBSMB-PAV can meet the S value and m-value requirement at 12 °C. Therefore, the low-temperature grade of SBSMB is PG-22 and the low-temperature continuous grade is −23.64 °C. Following the same analysis, the low-temperature grade of SBS/DCRMB is PG-28 and the low-temperature continuous grade is −28.59 °C. The main reason for this is that the SBS and DCR formed a three-dimensional network; when the strain reached the limit, the fracture stress can be rapidly concentrated on the surface of DCR. Then, DCR absorbs and consumes the energy, which prevents the formation and expansion of cracks, increasing low-temperature creep deformation. After thermal-oxygen aging and pressure aging, the original bitumen developed brittle and rigid characteristics and the polymer network broke down with the degradation and condensation. These effects further increase the S value and decrease the m-value. Comparing the decay laws of low-temperature properties for different aging conditions shows that the growth of the stiffness modulus and the creep rate of SBSMB before and after pressure aging both exceed that of SBS/DCRMB.

### 3.6. Analysis of and Anti-aging Properties

To some extent, the sensitivity of the asphalt binder’s performance affected by aging is revealed by the variation amplitude of the rheological performance and chemical composition indices of asphalt binder before and after aging, which are also important to evaluate the anti-aging performance of asphalt binder. These aging indices were presented in Equations (9)–(12) [49,50].

(9) $$MAI=\frac{\left|\mathrm{Unaged}\;\frac{\mathrm{Mn}}{\mathrm{Mw}}-\mathrm{Aged}\;\frac{\mathrm{Mn}}{\mathrm{Mw}}\right|}{\mathrm{Unaged}\;\frac{\mathrm{Mn}}{\mathrm{Mw}}}\times 100\%$$

(10) $$JDAI=\frac{\left|\mathrm{Unaged}\;J_{\mathrm{nr}-diff}-\mathrm{Aged}\;J_{\mathrm{nr}-diff}\right|}{\mathrm{Unaged}\;J_{\mathrm{nr}-diff}}\times 100\%$$

(11) $$N_{f}AI=\frac{\left|\mathrm{Unaged}\;N_{f}-\mathrm{Aged}\;N_{f}\right|}{\mathrm{Unaged}\;N_{f}}\times 100\%$$

(12) $$T_{L}AI=\frac{\left|\mathrm{Unaged}\;T_{L}-\mathrm{Aged}\;T_{L}\right|}{\mathrm{Unaged}\;T_{L}}\times 100\%$$

According to the above definition of the aging index, the sensitivity of the SBSMB and SBS/DCRMB to aging in different aspects of various temperature rheological performance and chemical composition is listed in Table 4. It can be seen that aging sensitivity of SBSMB and SBS/DCRMB is different in the total temperature domain.

According to the above definition of the aging index, the sensitivity of the SBSMB and SBS/DCRMB to aging in different aspects of various temperature rheological performance and chemical composition are listed in Table 4. It can be seen that aging sensitivity of SBSMB and SBS/DCRMB is different in the total temperature domain, but all aging indexes of SBSMB are larger than that of SBS/DCRMB in both short-aging and long-aging at the macro and micro levels, SBSMB is approximately twice that of SBS/DCRMB. It indicated that SBS/DCRMB possesses a better anti-aging ability due to absorption of lightweight components by composite modifiers (SBS and DCR) and release of carbon black from DCR.

## 4. Conclusions

The objective of this study was to evaluate the modification mechanism of SBS/DCRMB and investigate the evolution of the resulting rheological properties before and after TFOT and PAV aging by using fluorescence microscopy, GPC, DSR, and BBR. These experimental methods provide multi-scale data identifying the relationship between microstructure and macroscopic rheological properties. The specific benefits of these testing methods are listed in the following.

(1) The fluorescence micrographs confirmed that the DCR and SBS modifiers in SBS/DCRMB had been vulcanized and produced a three-dimensional network structure under the action of sulfur. Moreover, using GPC to analyze the molecular distribution of SBS/DCRMB shows only two regions (residual asphalt and polymer), which further indicates that a crosslinking reaction occurred between the polymers of SBS/DCRMB.
(2) GPC testing results indicated that the introduction of DCR in SBS/DCRMB significantly improved the thermal oxygen anti-aging ability, and the results of the LAS and MSCR test also indicated the same pattern.
(3) The MSCR testing results illustrated that DCR could effectively improve the elastic properties and decrease the viscous parts of asphalt. Furthermore, the three-dimensional polymer network structure formed by DCR and SBS plays a significant role in the high-temperature properties.
(4) DCR enhanced the resistance to fatigue cracking of SBS/DCRMB and combination with the GPC test results, shows that the influence of aging on the fatigue life of SBSMB was mainly the result of the hardening of residual asphalt. In contrast, the fatigue performance loss of SBS/DCRMB was primarily caused by the condensation of the polymer part and the disintegration of the polymer network structure.
(5) According to the stiffness modulus and creep rate results of the BBR test, the temperature sensitivity and anti-aging properties of SBS/DCRMB were significantly better than those of SBSMB.

## Figures and Tables

**Table 1:** Basic properties of bitumen.

Properties	Unit	Test Results			Test Method
		Neat	SBS	SBS/DCR	
Penetration (25 °C, 100g, 5 s)	(0.1 mm)	68.9	58.9	72.4	ASTM D5 [30]
Softening point (ring and ball method)	°C	47.2	66.5	85.8	ASTM D36 [31]
Ductility (15 °C, 5 cm/s)	Cm	>100	--	--	ASTM D113 [32]
Ductility (5 °C, 5 cm/s)	Cm	--	20.4	44.5	ASTM D113 [32]
Change in mass TFOT	%	−0.2	−0.11	−0.08	ASTM D2872 [33]
Flashpoint, Cleveland open cup	°C	289	291	310	ASTM D92 [34]

**Table 2:** Molecular weight calculation of SBSMB and SBS/DCRMB before and after aging.

Binder Type	Aging Conditions	M_n_	M_w_	M_n_/M_w_
SBSMB	Virgin	850	2270	2.67
	TFOT	885	2303	2.60
	PAV-20 h	928	2354	2.54
SBS/DCRMB	Virgin	860	2577	3.00
	TFOT	884	2664	3.01
	PAV-20 h	910	2790	3.07

**Table 3:** Linear amplitude sweep test results of all tested binders based on viscoelastic continuum damage analysis.

Binder Type	Aging Conditions	C_1_	C_2_	A	B	α	$\tau_{\max}$
SBS	Virgin	0.050	0.473	3,142,334	2.894	1.447	0.236
	TFOT	0.059	0.496	1,856,421	2.888	1.444	0.258
	PAV-20 h	0.066	0.521	1,533,497	2.922	1.461	0.318
SBS/DCR	Virgin	0.058	0.490	8,010,102	2.978	1.489	0.199
	TFOT	0.054	0.504	5,165,232	3.026	1.513	0.210
	PAV-20 h	0.050	0.513	3,456,893	3.042	1.521	0.249

**Table 4:** Aging sensitivity of two modified bitumen.

Modified Asphalt	MAI, %	JDAI, %	T_L_AI, %	N*_f_*AI, % (Strain = 1%)
SBSMB-TFOT	2.63	12.13	11.67	45.7
SBS/DCRMB-TFOT	0.33	6.75	5.72	14.0
SBSMB-PAV	4.87	32.67	56.63	80.6
SBS/DCRMB-PAV	2.33	20.47	28.4	63.0
